# Blood glucose variability, measured as mean absolute glucose, strongly depends on the frequency of blood glucose level measurements

**DOI:** 10.1186/cc9812

**Published:** 2011-03-11

**Authors:** RE Harmsen, F Van Braam Houckgeest, PE Spronk, MJ Schultz, A Abu-Hanna

**Affiliations:** 1 Academic Medical Center, Amsterdam, the Netherlands; 2 Tergooi Hospitals, Hilversum, the Netherlands; 3 Gelre Hospitals, Apeldoorn, the Netherlands

## Introduction

Blood glucose variability (BGV) has been associated with outcome of critically ill patients [1-3]. Different BGV metrics exist, including mean absolute glucose (MAG) [4], which is the mean of absolute change per hour in glucose level. We hypothesized MAG to depend on the blood glucose level (BGL) measurement frequency, as doing more measurements could lead to more changes in the insulin infusion rate and hence in changes in the follow-up BGL.

## Methods

We developed and implemented an evidence-based guideline for intensive insulin therapy on a mixed medical-surgical ICU in the Netherlands. The new guideline explicitly specifies when the follow-up BGL measurements should be taken, and hence influences BGL measurement frequency. We collected all BGL measurements, patient demographics and outcome information for 1 year before and 1 year after the guideline's implementation, and analyzed the association of MAG and mortality.

## Results

Data for 758 and 601 patients were collected 1 year before and 1 year after implementation. The two cohorts had similar baseline characteristics: median age 71 (59 to 80) years, median APACHE II scores 17 (13 to 23). Hospital mortality did not change (30.7% and 31.6%, *P *= 0.729). After implementation, median BGL decreased from 117 (97 to 144) to 106 (90 to 130) mg/dl (*P *< 0.001), and the median BGL measurement frequency doubled, from 4 (3 to 6) to 8 (4 to 11) per day per patient (*P *< 0.001). MAG increased from 4.5 (2.5 to 7.0) to 6.6 (3.6 to 9.7) mg/dl/hour (*P *< 0.001). Both BGL measurement frequency and the APACHE II score significantly correlated with the MAG (Pearson's correlation coefficient 0.574 and 0.19, respectively). The MAG was not independently associated with mortality when adjusting for both measurement frequency and the APACHE II score (odds ratio 1.01 (0.98 to 1.05), *P *= 0.42).

## Conclusions

The association between MAG and BGL measurement frequency and severity of illness requires careful interpretation when comparing cohorts differing in BGL measurement frequencies. It also requires adjustment for these variables when investigating the association between MAG and mortality, as it did not emerge as an independent predictor in our cohort.
